# Modern Medico-Psychology and Psychiatry

**Published:** 1894-12-22

**Authors:** T. S. Clouston

**Affiliations:** Lecturer on Mental Diseases, Edinburgh University, and Physician Superintendent Royal Asylum, Morningside


### Modern Medico-Psychology and Psychiatry.

By T. S. Clouston, M.D., Lecturer on Mental Diseases, Edinburgh University, and Physician Superintendent Royal Asylum, Morningside.

(*Continued from page 4.*)

### XIII.—States of Defective Control and of Morbid and Uncontrollable Impulse.

There is no satisfactory test or definition of legal or of medical "insanity," but the nearest approach to such a test and definition is—"A loss of mental and nervous inhibition from brain disease." Disordered intelligence, altered emotion, lost memory, are all in a certain degree more consistent with legal and technical "sanity" than diminished control over speech and action, with morbid impulses towards suicide or homicide and certain anti-legal or anti-social actions due to brain disease. Perfect control is impossible in any man, but a certain degree of control is requisite for sanity and citizenship in all men. All legal enactments, all ethical codes, all social arrangements imply a certain freedom, volition, and action. Responsibility to this law, to society, and to God is founded on this assumption. All blame and all punishment imply a certain minimum at least of self-control. Yet the power of control is a quality of brain just as motion and sensation are brain qualities. It is absent in early childhood. The baby of six months has no endowment that can be called mental inhibition. It is developed as the brain grows. There are half and quarter stages of control as there are of muscular co-ordinations. The law and society admit this, for legal responsibility for crime does not begin till a certain age has been attained. In some children this inhibitory faculty is stronger and soon developed; in others it is of slow growth. It does not bear a fixed relationship to the other mental or bodily faculties. A boy at twelve may be intelligent and strong, yet his faculty of control may be very weak indeed or almost absent. There may be arrested development of the controlling faculties, while the other mental faculties may be normal. A lad of sixteen may be a good reader, may write well, may know some sciences, geography, and arithmetic, yet may commit murder or incendiarism or sexual offences, for which he is irresponsible because the faculty of control was absent. There are families of criminals in whom the power of control over action is congenitally absent, just as there are families who never develop beards. Its complete or partial absence is hereditary. Successive members of these families at early ages become drunkards, prostitutes, thieves, and murderers automatically, as it were, irrespective of design and "motive." My own experience and the testimony of innumerable competent medical observers leave no sort of room for doubt that these are facts, and not mere medical theories.

Passing into the region of morals and duty, I have no sort of doubt, from my experience, that the subjective sense of right and wrong, as well as the power to follow the one and avoid the other, depends on brain as well as on education. There are some persons born with brains so constituted that they have no conscience, no moral instincts, and no remorse when they have done wrong. No amount of instruction, no force of example will rouse in them the "inward monitor," just because its brain vehicle does not exist in them. You might as well blame a colour-blind railway signalman for sending a train on to destruction as blame them for moral callousness or actual crime. This is not the place to enter on the question of what should be done with such persons, or whether they should be held responsible by the law. The sciences of physiology, psychology, and sociology need to advance several steps before we can alter the present laws. We must first convince the public of the facts, and make it understand their import, before we can reasonably expect that long-standing arrangements that suit ninety-nine cases out of a hundred can be altered for the sake of the hundredth. The public must be fully satisfied before laws that seem of universal application, and absolutely necessary for society, are changed.

Apart from law and ethics, the study of the dissolutions of control in insanity has the greatest interest, and is of primary importance. As a matter of fact, inhibition, nervous or mental, is lost in most cases of mental diseases sooner than judgment or memory or emotion. Patients are conscious of a loss of control over their thoughts or imaginations, or they have impulses towards morbid acts before they become depressed or exalted, or incoherent, or express delusions as they do in many cases of melancholia and in most cases of mania. Few men or women become tired or pass through influenza or any other more exhausting diseases, or indulge in excesses without losing much of their normal power of control, or without having morbid impulses to do acts which intellectually they see to be foolish or wrong. We know there exists, lying deep down in the nature of most human beings, potentialities of thoughts that are not reasonable, of actions that are not conventional, or social, or moral. The "animal" has certainly not been thoroughly evolved out of men, and when the mental brain cortex is becoming exhausted or starved, or diseased as in incipient insanity, along with loss of control over thought, there are often enough impulses towards killing, towards unnatural sexual practices, towards wanton destructiveness, towards appropriation for self of what belongs to others. And there are frequently enough uncontrolled or uncontrollable actions, shouting, grimacing, dancing, talking, &c., in secret long before the man is legally insane. The great and almost constant preliminary symptom of an attack of insanity, sleeplessness, I believe to be of this nature of a loss of inhibition. The normal controlling centres of the blood vessels of the brain, by the action of which the blood supply is shut off to a large extent from the mental cortex before sleep comes on, are weakened so that a congestion instead of an anæmia is produced, and no sleep can therefore be obtained. Insomnia, if this view be correct, is therefore the first and the most important of all the losses of normal inhibition in ordinary insanity.

Physiologists have been able to define "inhibitory centres" in the medulla cord and brain cortex through the action of which muscular action is quickened or controlled, blood vessels are enlarged or contracted, and other nerve centres are put into action or restrained from action. This fact, together with Laycock's law of cerebral reflex action or cerebral automatism, whereby the brain may do complex actions without any volition or purpose, helps us to explain many of the impulsive actions of insanity and allied conditions. I shall reserve for another article a more detailed description of the various forms of insane impulse and uncontrollable action.

